# Intraoperative Monitoring of Sensory Evoked Potentials in Neurosurgery: A Personalized Approach

**DOI:** 10.3390/jpm15010026

**Published:** 2025-01-13

**Authors:** Evgeny A. Levin

**Affiliations:** E.N. Meshalkin National Medical Research Center, Ministry of Health of the Russian Federation, 15 Rechkunovskaya St., Novosibirsk 630055, Russia; e_levin@meshalkin.ru

**Keywords:** intraoperative neurophysiological monitoring, somatosensory evoked potentials, visual evoked potentials, brainstem acoustic evoked potentials, signal-to-noise ratio, frequency filters, stimulation rate, personalized approach

## Abstract

Sensory evoked potentials (EPs), namely, somatosensory, visual, and brainstem acoustic EPs, are used in neurosurgery to monitor the corresponding functions with the aim of preventing iatrogenic neurological complications. Functional deficiency usually precedes structural defect, being initially reversible, and prompt alarms may help surgeons achieve this aim. However, sensory EP registration requires presenting multiple stimuli and averaging of responses, which significantly lengthen this procedure. As delays can make intraoperative neuromonitoring (IONM) ineffective, it is important to reduce EP recording time. The possibility of speeding up EP recording relies on differences between IONM and outpatient clinical neurophysiology (CN). Namely, in IONM, the patient is her/his own control, and the neurophysiologist is less constrained by norms and standards than in outpatient CN. Therefore, neurophysiologists can perform a personalized selection of optimal locations of recording electrodes, frequency filter passbands, and stimulation rates. Varying some or all of these parameters, it is often possible to significantly improve the signal-to-noise ratio (SNR) for EPs and accelerate EP recording by up to several times. The aim of this paper is to review how this personalized approach is or may be applied during IONM for recording sensory EPs of each of the abovementioned modalities. Also, the problems hindering the implementation and dissemination of this approach and options for overcoming them are discussed here, as well as possible future developments.

## 1. Introduction

Intraoperative neurophysiological monitoring (IONM) has been used in neurosurgical operations to prevent iatrogenic postoperative neurological complications for more than 40 years [1] and has become increasingly widespread in the last two decades. The registration of sensory evoked potentials (EPs)—somatosensory (SSEPs), visual (VEPs), and brainstem acoustic (BAEPs)—is used to check on corresponding sensory functions during brain surgeries performed under general anesthesia. In all these cases, the stimuli of certain modalities are presented to a patient, and the responses to them are recorded from the corresponding areas of the cerebral cortex, brainstem structures, or peripheral elements of the nervous system. The responses obtained at the beginning of the surgery are used as a baseline, and those obtained during the operation are compared with them. If signs of intraoperative deterioration of the monitored function appear (these are, first of all, a decrease in the amplitude and/or an increase in the latency of certain peaks in EP curves), the neurophysiologist informs the surgeons about it, and the latter, if possible, take actions to protect the affected neural structure.

Sensory EPs have two fundamental drawbacks that can substantially diminish their effectiveness in preventing iatrogenic neurological complications. First, similar to other monitoring methods, they detect a dangerous situation only when the dysfunction has already begun. Fortunately, in many cases, functional deficiencies precede a structural defect and are reversible at an early stage. It partially compensates for this drawback; however, a critical prerequisite for this compensation is to promptly inform surgeons about the signs of the onset of dysfunction. And, at this stage, the second drawback becomes apparent: the registration of sensory EPs can be quite time-consuming. This is due to the fact that the amplitudes of the measured neural responses to the sensory stimulation are typically one to two orders of magnitude lower than the background (unrelated to stimuli) activity of the nervous system, which can be regarded as noise in the context of EP recording. Furthermore, electrophysiological signals recorded in the operating room may be significantly affected by external electromagnetic noise. A classic approach to addressing the issue of a small signal-to-noise ratio (SNR) is to use averaging: the same stimulus is presented repeatedly, and response segments time-locked to the moments of stimulus presentation are summed and averaged. The average value for stimulus-unrelated activity tends to zero with an increasing number of averages, whereas responses time-locked to stimuli are preserved during averaging. The required number of averages depends on the SNR and, according to the existing recommendations for recording sensory EPs, is up to 100–200 in the case of SSEPs [2] and VEPs [3] and up to 1000–2000 in the case of BAEPs [4,5]. The stimulation rate is constrained by the properties of the nervous system, resulting in EP recording durations that vary from tens of seconds to several minutes. Additionally, it is often necessary to record several EPs of the same modality (for example, when stimulating the right and left sides), and multimodal monitoring is also a common option. Consequently, the intervals between consecutive records of sensory EPs may be too prolonged, thereby creating the risk of providing surgeons with too delayed feedback and, accordingly, reducing the effectiveness of monitoring.

However, this problem could be solved by identifying methods to increase the SNR, which would reduce the required number of averages and allow for controlling sensory EPs more often. And such methods do exist because, as noted above, in IONM, the patient serves as her/his “own control”. This allows neurophysiologists to use a personalized approach to the selection of the parameters of signal recording and signal processing methods, as well as to vary the characteristics of the presented stimuli. Indeed, while in conventional clinical neurophysiology (CN), the task is to compare EPs obtained from the patient with the normative ones, in IONM, the task is different—to detect new changes in EPs if they appear during the surgery. In the first case, the recording and processing of EPs must adhere to the same parameters as when obtaining normative data. In the second, these parameters may vary between monitoring sessions: it is only necessary that the recorded responses are clearly distinguishable and that their changes are readily detectable. This and other differences between the registration of sensory EPs in outpatient settings and their intraoperative monitoring are summarized in Table 1.

This review examines several methods for increasing the SNR for SSEP, BAEP, and VEP recording during brain surgery, namely, the personalized selection of recording electrodes localization, frequency filter passband, stimulation rate, and stimulus characteristics. It provides theoretical justifications for using these methods and examples of the practical implementation of a personalized approach to sensory EP monitoring. Furthermore, factors that may hinder the implementation of a personalized approach to the intraoperative monitoring of sensory EPs are analyzed and options for overcoming these problems are proposed as well as possible future developments.

As a concluding introductory remark, it is important to acknowledge that a certain degree of personalization is inherent in all clinical neurophysiology methods, including evoked potentials, not only in IONM but also in outpatient settings. It is not unusual to make slight adjustments to the recording electrode position or raise the HPF cutoff frequency from 1 to 2 or even 5 Hz to remove baseline wandering and/or high-amplitude delta activity. However, IONM conditions provide significantly greater flexibility in selecting parameters for the recording and processing of EPs. The differences between, for example, moving the reference electrode from Fz to FCz and changing its position from Fz to the mastoid, or raising the HPF cutoff from 1 to 2 Hz and from 1 to 15 Hz, are rather qualitative than quantitative. However, such substantial modifications to standard neurophysiological techniques can often lead to no less significant acceleration in the acquisition of sensory EPs. This, in turn, contributes to achieving the primary objective of neuromonitoring: protecting the quality of life of the patients.

## 2. Key Parameters for Optimizing Intraoperative Monitoring of Sensory Evoked Potentials

As noted above, one of the main problems in the intraoperative monitoring of sensory EPs, which reduces its effectiveness, is the necessity to use the averaging method, which prolongs their registration. The possibility of optimizing the parameters of sensory EP registration for their faster acquirement was first demonstrated by J. Fridman et al. [6]. They showed that the optimal frequency filter selection for BAEP registration led to a significant increase in the SNR and a several-fold decrease in the number of averages required to obtain reproducible responses. A personalized approach to neuromonitoring parameters selection (in this case, to the choice of recording electrodes’ locations for the intraoperative monitoring of SSEPs), which was also called “optimization”, was proposed by D.B. MacDonald [7]. The search for the most effective parameters for sensory EP monitoring represents a specific instance of the classical optimization problem with a multidimensional parameter space, a set of boundary conditions, and an objective function. The primary parameters to be optimized include the localization of the recording electrodes, the passband of the frequency filter, the stimuli presentation rate, and the characteristics of the stimuli themselves. In this context, the objective function is the duration of EP recording (minimized for a given target SNR). If optimal frequency filter settings are selected using previously recorded EPs (unfiltered data should be used for such selection), the SNR of recorded EPs after filtration may serve as a surrogate objective function.

***Localization of recording electrodes.*** Since EPs are a type of bioelectrical activity, their registration requires measuring the difference in electrical potential between two electrodes. The location of one of these electrodes is typically predetermined by the localization of the area in which the activity is being monitored; it is usually positioned as close as possible to this area of interest and is called active. The second electrode, the reference, relative to which the potential on the active electrode is measured, is commonly located in the area with minimal activity. In the case of sensory EPs of cortical origin (VEPs, cortical SSEPs), their sources, in many cases, can be adequately represented using the equivalent dipole model [8]. In this situation, positioning the reference electrode at the projection of the opposite pole of the dipole may yield higher signal amplitudes than using the “zero” reference. Moreover, if the orientation of the equivalent dipole is tangential to the cranial surface, the optimal placement of not only the reference but also the active electrode can be quite far from the true source of activity. Importantly, factors such as individual neuroanatomical features, the impact of existing pathology, differential effects of anesthetics, and brain shift, which may occur during surgery, lead to significant variability in the optimal placings of the electrodes among patients, raising the task of their personalized selection. Also of note is that changes in volume conductivity associated with incision, trepanation, and opening of the dura mater can significantly change the spatial distribution of the potentials on the scalp, especially if an intracranial air gap develops at the cortical projection zone of the monitored sensory modality. In this regard, the final choice of the optimal localization of recording electrodes should be made after opening the dura mater. During brain surgeries, the head surface is usually inaccessible for placing new electrodes, so all electrodes intended for optimal combination selection should be installed during the preoperative preparation stage.

***Passband of the frequency filter.*** The frequency filter is a powerful instrument that effectively eliminates activity with irrelevant frequency characteristics when registering EPs; this tool is employed, in most cases, during sensory EP registration [2,9,10]. The recommended passband of the frequency filter for registering EPs in outpatient settings is typically quite wide since, in this case, it is important to compare the undistorted signal characteristics with those of normative data. In the case of IONM, the patient serves as her/his own “control” and even if filtering distortions are present, they equally affect both the baseline and subsequently obtained data. Provided that the EP peaks remain discernible, such distortions generally do not hinder the feasibility of detecting changes in their latencies and amplitudes. This allows for a more “aggressive” filtering approach with a significantly narrowed filter passband. However, narrowing the passband not only distorts the shape of EPs but also reduces the amplitudes of their peaks, thereby creating a trade-off between the attenuation of noise and signal amplitude reduction. At the same time, the frequency spectra of both the EPs themselves and the activity regarded as noise during EP registration (true noise and background EEG and EMG) vary among patients and are subject to the significant influence of anesthetics. Thus, a personalized approach is necessary to select optimal frequency filter parameters for the intraoperative monitoring of sensory EPs.

***Stimuli presentation rate.*** For each modality of sensory EPs, the existing recommendations suggest using its own stimuli presentation rate: from about 1 Hz for VEPs [10] to 2–5 Hz for SSEPs [2] and 10–15 Hz for BAEPs [4]. In order to minimize the time required for recording EPs, it would be desirable to present stimuli as frequently as possible. However, increasing the stimuli presentation rate is constrained, firstly, by the need to avoid the overlapping of responses to successive stimuli, and, secondly, by the phenomenon of attenuation of responses when stimuli are delivered at excessively high rates. The first factor rigidly limits the presentation rate of visual stimuli to about 3 Hz, somatosensory stimuli to 20 Hz for stimulation of the upper limbs and 15 Hz for stimulation of the lower limbs, and acoustic stimuli to 100 Hz. The second factor introduces another trade-off: beyond a certain frequency, the effect of decreasing the amplitudes of the EPs outweighs the effect of increasing the stimulation frequency. However, for BAEPs and SSEPs, there are studies [11,12] indicating that the optimal stimulation frequency for accelerating EP recording may exceed the values given in clinical recommendations, including that for IONM. There is also some room for a personalized approach regarding this parameter: the relation between the increase of the stimulation rate and the attenuation of the EPs varies among patients and depends not only on individual characteristics and the effects of existing dysfunctions but also on the depth of anesthesia, body temperature, etc. As an additional note, it should be mentioned that if artifactual oscillations with a fixed frequency are observed during the record, a minor (a few percent) adjustment in the stimulation rate in any direction may significantly enhance the recording quality [13]. The power supply network interference is a fixed frequency artifact that is almost always present, having a frequency of 50 or 60 Hz depending on the country. Therefore, when selecting the optimal stimulation frequency, values that are submultiples of the network frequency should be avoided.

***Characteristics of the stimuli.*** Modifications of stimuli can be employed to enhance the amplitude of evoked responses or, in some cases, to reduce stimulation artifacts. In the case of SSEPs, the personalized selection of stimulation intensity is widely regarded as a standard practice; for example, ISIN recommendations [14] suggest using so-called supramaximal stimulation estimated as twice the motor threshold for each stimulation point. This seemingly excessive intensity is required to prevent false alarms that may arise from clinically insignificant fluctuations in the current reaching the stimulated nerve. Apart from that, standard constant-current rectangular 0.2–0.3 ms pulses are typically highly effective in producing clear SSEPs and hardly require modifications. In contrast, for VEPs and BAEPs, some non-standard stimulation methods have been described; however, these methods remain largely experimental. A more detailed discussion of these techniques will be presented in the relevant sections.

Thus, for all four parameters considered, their personalized optimization is possible to minimize the time required to record EPs with a given SNR or to maximize the SNR of the obtained EPs under given time constraints. Below, the options for this optimization will be discussed in more detail for each of the three modalities of sensory EPs used in IONM.

## 3. Somatosensory Evoked Potentials

SSEPs have a long history of use in IONM and are the most widely used sensory EPs for IONM [2]. This prevalence likely accounts for the advanced development of their monitoring methodology, including the issue of its personalization. The latter can be largely attributed to the merits of D.B. MacDonald, who has been promoting the idea of the optimization of (de facto personalized approach to) SSEP monitoring for more than two decades [7,14,15,16,17]. In his initial publications in this field [7,15], optimization concerned the optimal choice of derivations for recording cortical SSEPs from the lower extremities. It was shown that, firstly, the then-recommended derivation CPz–Fpz* was not optimal for any patient, and, secondly, several variants were preferred, which could vary between individual patients and sometimes even between different stimulation sides in the same patient. The optimal derivations most frequently were Cz–CPc and CPz–CPc; Pz–CPc, CPi–CPc, and some other variants were also optimal for a smaller subset of patients. Compared to the CPz–Fpz derivation, the optimal variants resulted in an average increase in the SNR by more than twofold and a decrease in the required number of averages by a factor of four.

(*—From here on, when considering SSEPs and BAEPs, the locations of the electrodes along the fronto–occipital axis are designated in accordance with the 10–10 system [18]. For electrodes along the midline, the designations are completely consistent with this system (e.g., CPz). For electrodes located lateral to the midline, instead of numerical values encoding the side (odd numbers—left side, even—right) and distance from the midline (a higher number corresponds to a greater angular distance), the designations i and c (e.g., CPi and CPc) will be used, meaning the hemisphere ipsi- and contralateral to the stimulation side, respectively. Here, they correspond to electrodes with numerical codes of 3 or 4 (20% distance from the midline). In the VEP section, all electrode location designations are the same as in [18].)

Neuroanatomically, the superiority of the “transverse orientation” of leads over the “longitudinal orientation” when recording cortical SSEPs from the lower extremities is well-founded: the cortical representation of the foot is located in the interhemispheric fissure [19], and the axis of the corresponding equivalent dipole [8] is oriented across its plane tangentially to the surface of the skull. Accordingly, in the “transversely oriented” leads, the two electrodes are positioned in zones exhibiting the opposite polarities of electric potential, and in the “longitudinally oriented” leads—near the equipotential line. For SSEPs from the upper extremities, this “dipole effect” is less pronounced since the cortical representation of the hand is located on the convexity, and at least some of the sources of these SSEPs generate a potential equivalent to a radially oriented dipole located close to the skull [8]. As a result, the distribution of potentials on the scalp varies among patients to a lesser extent. Accordingly, the study by MacDonald et al. [16] showed that, in most cases, the CPc–CPz derivation was optimal, which they recommended to adopt as the standard. At the same time, in some patients, the CPc–Fz and CPc–CPi derivations occurred optimal and were suggested as possible alternatives. The current ISIN clinical guidelines [14] advocate for these derivation selection strategies when monitoring cortical SSEPs from the lower and upper extremities. In a more recent position statement by the ACNS [2], the localization of active electrodes aligns with that proposed by MacDonald, and the question of reference electrode localization is omitted, leaving the neurophysiologist free in their choice even if she/he is obliged to follow published standards.

Since the personalized approach entails the pre-emptive placement of recording electrodes in several locations (see the previous section) if the equipment used has a sufficient number of channels, it becomes possible to record SSEPs simultaneously from several derivations. The advantage of utilizing multichannel EPs lies in the capacity to avoid false alarms in cases when signs of dysfunctions in SSEPs are observed solely in one of the derivations while the remaining SSEPs do not change significantly [20]. This scenario may occur, for example, when a local intracranial air gap develops adjacent to the resection zone after the removal of a large volume of pathological tissue.

It should be noted that in the above reasoning, the SNR was determined, in fact, by solely the amplitude of the SSEPs itself (signal), and the noise factor, both from the background EEG and myographic, was ignored. For the intraoperative monitoring of cortical SSEPs, this approach is deemed to be acceptable since when recording them, it is permissible to use a high-pass filter (HPF) up to 30 Hz and a low-pass filter (LPF) of about 300–500 Hz, as it is directly indicated in current clinical guidelines [2,14]. Such filtering effectively suppresses high-amplitude “EEG noise” in the alpha range and significantly reduces the amplitude of EMG artifacts. Consequently, when employing this filter, it is often possible to select the localization of the SSEP recording electrodes without taking these noise factors into account. At the same time, the range of 30–300 Hz is sufficiently broad to encompass, in most cases, the frequencies corresponding to the SSEP waves analyzed during monitoring [21]. In this situation, there may be no necessity to customize a personalized frequency filter passband for SSEPs. However, in cases where significant pathological effects or excessive anesthesia depth are present, SSEPs can slow down considerably, which may necessitate a reduction in the lower passband edge, especially for SSEPs from the lower extremities. Additionally, when using a high stimulation rate (see below) together with interleaving stimulation, the stimulation artifact from the subsequent stimulated limb may overlap with the response from the previous one. In this case, it may be necessary to elevate the upper passband edge so that these sharp high-amplitude artifacts are clearly distinguished from genuine SSEPs.

The possibility of varying the stimulation rate when monitoring cortical SSEPs is discussed in the recent position statement by ASNM [2]. It is proposed to use a stimulation rate of 2 to 5 Hz for SSEPs from the lower limbs, with the possibility of increasing it to 9 Hz for SSEPs from the upper limbs. The possibility of improving the quality of responses by using a stimulation rate near the lower limit was also mentioned, especially for cases with pre-existing dysfunctions [17]. In a recent study by Dimakopoulos et al. [12], a direct comparison of the SNR achieved for a fixed recording time at different stimulation rates was performed for cortical SSEPs from the upper and lower extremities. For the upper extremities, it was shown that when the recording duration was limited to 5 s, the maximum SNR (median value of about 22) for the N20 peak was achieved at a stimulation rate of 12.7 Hz, and for 10 and 20 s, at a stimulation frequency of 4.7 Hz (median SNR values of about 35 and about 55, respectively). For the lower extremities, a stimulation frequency of 4.7 Hz emerged as optimal across all recording durations, yielding median SNR values for the P40 peak of about 20, 30, and 35 at 5, 10, and 20 s of recording, respectively. These findings were derived from group-level analysis, and the interindividual variability was very large (e.g., the interquartile range for the SNR for the N20 peak at 12.7 Hz stimulation was about 20 with a median of about 22). Consequently, these values should not be regarded as definitive recommendations for individual cases. However, they can be used as a starting point for the personalized selection of the stimulation rate. Of note, the proposals to increase the stimulation rate are met with some objections, citing the fact that a decrease in SSEP amplitudes is already observed when transitioning from 1 to 3 Hz, which continues with a further increase in the stimulation rate [22]. However, the data presented in this publication show that the amplitudes of the SSEP peaks for the stimulation rates from 1 to 5.5–8.5 Hz decrease to a much lesser extent than the increase in rate, which makes this increase justified for the aim of rapidly obtaining well-reproducible responses.

In general, intraoperative SSEP monitoring has seen significant advancements in the personalized optimization of its parameters. The current clinical recommendations explicitly or implicitly acknowledge the potential for such optimization [2,14]. The integration of optimization techniques with interleaved stimulation enables the rapid acquisition of SSEPs from all four extremities, often in less than one minute. This rapidity facilitates prompt communication with surgeons regarding the emergence of dysfunction signs, thereby enhancing the overall effectiveness of monitoring [17].

## 4. Visual Evoked Potentials

For VEPs, IONM standards have not been established yet. The existing literature presents a variety of methods for their registration, which differ significantly in terms of electrode localization, frequency filter ranges, and the number of averages utilized in VEP registration. Some of these publications and the parameters of intraoperative VEP recording addressed in them are listed in Table 2. Notably, almost none of these works provide a rationale for the choice of these parameters. The following discussion will examine the advantages and disadvantages of various options for each of these parameters and propose an algorithm for their selection aimed at the personalized optimization of intraoperative VEP monitoring.

**Figure 1:**
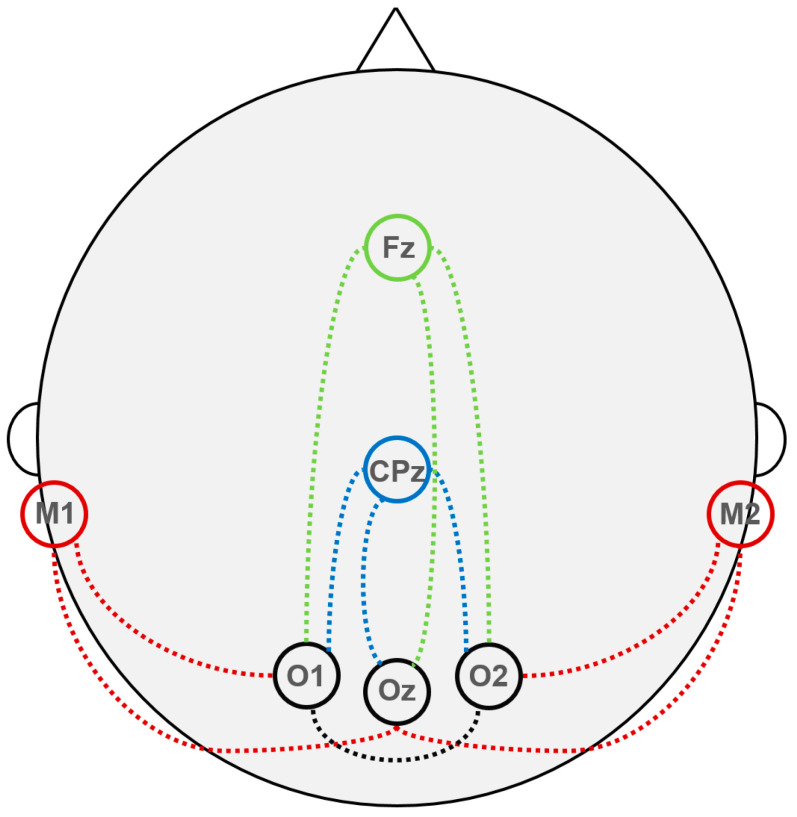
Possible electrode locations for visual evoked potential monitoring. The notation mostly follows the 10–10 EEG system [18]; M1 and M2 mean electrodes placed over the left and right mastoid process, correspondingly. Black circles—active electrodes over the occipital cortex (visual cortical area); colored circles—possible locations of the reference electrodes. Adapted from [37] “Intraoperative monitoring of visual evoked potentials: experience of 240 operations”, by E.A. Levin, M.G. Kilchukov and A.A. Glushaeva, 2024, *Neyrokhirurgiya = Russian Journal of Neurosurgery*, *26* (3), p. 59, Figure 1a (https://doi.org/10.17650/1683-3295-2024-26-3-57-71). CC BY 4.0 (https://creativecommons.org/licenses/by/4.0/, accessed on 20 November 2024).

When VEPs are recorded for clinical diagnostic purposes, it is generally recommended to place the reference electrode at the Fz position [10] of the 10–10 [18] EEG electrode placement system (Figure 1, green); the same placement is sometimes utilized for the intraoperative monitoring of these EPs [23,24,25]. However, under general anesthesia, the characteristics of the background bioelectrical activity can change significantly. In particular, during anesthesia with propofol, which is considered the preferred anesthetic for monitoring VEPs [26], anteriorization of the alpha rhythm occurs [27]. A similar effect was also observed in some patients under inhalation anesthesia with sevoflurane [28]. As a result, the reference electrode in the frontal region may become a source of high-amplitude “alpha EEG noise”, making it preferable to place reference electrodes in other areas where background EEG activity is lower. Indeed, alternative reference electrode placements have been documented on the earlobes (A1/A2) [25,26,29,30,31], on the mastoids (M1/M2) [32,33] (Figure 1, red), and posterior to the vertex (CPz) [34,35,36] (Figure 1, blue).

**Figure 2:**
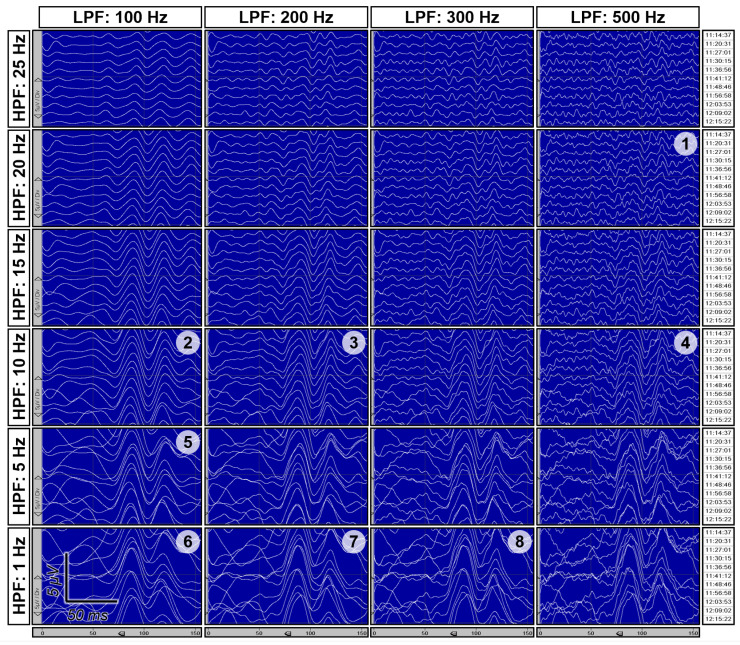
Results of applying filters with different high- and low-pass cutoffs to the same set of VEP recordings. Data from Oz–CPz derivation are presented. Differences in the reproducibility, amplitude, and shape of the VEP curves are clearly visible, as well as trade-offs between the amplitudes of VEP peaks and of noise. The numbers in the top right corners of some panels correspond to the papers listed in Table 2. These panels demonstrate the results of applying the same filter parameters as used in the following papers: 1—[32,33,38], 2—[25], 3—[36], 4—[26,29], 5—[3,24], 6—[24,39], 7—[30], 8—[35]. Note that the actual typical VEP curve shapes that were seen by the authors of the listed papers may differ to some extent from those presented here since some undocumented features of frequency filters (e.g., roll-off rate) can significantly affect the results of filtration. Adapted from [37] “Intraoperative monitoring of visual evoked potentials: experience of 240 operations”, by E.A. Levin, M.G. Kilchukov and A.A. Glushaeva, 2024, *Neyrokhirurgiya = Russian Journal of Neurosurgery*, *26* (3), p. 59, Figure 1b (https://doi.org/10.17650/1683-3295-2024-26-3-57-71). CC BY 4.0 (https://creativecommons.org/licenses/by/4.0/, accessed on 20 November 2024).

In our recent publication [37], we described a personalized approach to selecting the reference electrode for VEP recording. For this purpose, when preparing for monitoring, we installed possible reference electrodes at four positions, i.e., Fz, Cz, M1, and M2, and active electrodes at three, i.e., O1, O2, and Oz, in the left, right, and medial occipital regions (see Figure 1). Subsequently, we recorded VEPs two or three times prior to the beginning of the surgery, utilizing all reference electrodes configurations. After that, we selected combinations of electrodes for further monitoring based on the criteria of reproducibility, distinguishability, and amplitude of the peaks of the obtained VEPs while also considering various frequency filtering options (see below). A retrospective analysis of 240 surgeries in which VEP monitoring was performed using this approach showed that M1/M2 was selected in 47% of cases, Fz in 24%, and CPz in 23% (refer to Figure 1 for derivations shown in red, green, and blue, respectively). An additional 3% each involved combinations with an occipital reference electrode (derivations O1–O2/O2–O1, Figure 1, black) and cases in which VEPs could not be obtained for either the left or the right eye using any electrode combination. This variability may be attributed to the predominance of different types of noise across various patients and circumstances. On the one hand, in flash VEPs, the most reproducible and well-defined is the N2–P2–N3 complex, with latencies of the corresponding peaks of about 75, 110, and 150 ms [10] (p. 7, Figure 4), and these peaks are typically monitored in VEPs. These oscillations correspond to a frequency of about 15 Hz, and, consequently, the attempts to reduce the effect of “EEG noise” within the alpha range (8–13 Hz) by frequency filtering are often significantly constrained by the trade-off between noise suppression and the necessity to avoid significant suppression of the controlled response itself. This trade-off and other effects of applying different filters to VEP monitoring data are illustrated in Figure 2. On the other hand, reference electrodes in the temporal region (M1/M2/A1/A2) are susceptible to myographic artifacts from the temporal, masseter, and sternocleidomastoid muscles, particularly if the necessity to control motor functions (e.g., oculomotor) deters the use of muscle relaxants. It could seem that the best option should be CPz, located in an area devoid of cranial muscles and outside the zone of anteriorized alpha rhythm. However, in this case, the amplitude of the recorded VEPs may be reduced due to the relative proximity of CPz to the active electrodes. Thus, there is no unequivocally preferred option for localizing reference electrodes during VEP monitoring; rather, a personalized approach is essential to obtain the best SNR in each case. This necessity for personalization is particularly pronounced for VEPs but is equally applicable to the intraoperative monitoring of other sensory evoked potentials.

**Table 2:** Variability in intraoperative VEP recording parameters among different publications.

Publication	Locations of Recording Electrodes	Filter Passband	Figure 2 Number	Number of Averages	Stimulation Rate
Kodama et al., 2010 [29] *	P7/O1/Oz/O2/P8–(A1+A2)	10–1000 Hz	4 **	40–100	1/s
Sasaki et al., 2010 [32]	O1–M1, O2–M2	20–500 Hz	1	100	1/s
Scherbuk et al., 2011 [23]	O1/O2/Oz–Fz	2–100 Hz		200	1/s
Houlden et al., 2013 [24]	Oz–Fz	(1–5)–100 Hz	5, 6	150	1.41/s
Luo et al., 2015 [3]	O1/Oz/O2–Fz/Cz/(A1+A2)	5–100 Hz	5	100–200	ND
San-Juan et al., 2016 [40]	O1–O2, O1–Oz, O2–Oz, Oz–Fz	2–100 Hz		250	4.1/s
Sato, 2016 [30] *	P7/PO7/O1/Oz/O2/PO8/P8–(A1+A2)	0.1–200 Hz	7	100	1/s
Uribe et al., 2017 [38]	O1/Oz/O2–M1/M2/Fz	20–500 Hz	1	100	1.1/s
Gutzwiller et al. 2018 [34]	O1/Oz/O2–CPz	2–400 Hz		50	0.9/s
Hariharan et al., 2018 [35]	O1/Oz/O2–Cz	1–300 Hz	8	128	1.1/s
Houlden et al., 2019 [25]	Oz–Fz/(A1+A2)	10–100 Hz	2	50–100	1.41/s
Miyagashima et al., 2019 [33] *	O1/Oz/O2–M1/M2	20–500 Hz	1	100	1/s
Kodama, Goto, 2020 [26] *	P7/O1/Oz/O2/P8–(A1+A2)	10–1000 Hz	4 **	40–100	1/s
Nakagawa et al., 2022 [31]	O1/Oz/O2–(A1+A2)	ND		100	1/s
Olmsted et al., 2023 [36] *	O1/Oz/O2/PO1/POz/PO2–CPz	10–200 Hz	3	≥50	1.05/s
Tao et al., 2024 [39]	O1/Oz/O2–unspecified reference	1–100 Hz	6	<50	0.7–1.2/s

The data presented in the table are derived from the Methods sections of the corresponding papers. In the “Locations of recording electrodes” column, the designations preceding the dashes correspond to active electrodes, while those following the dashes refer to reference electrodes. The slashes (/) mean that multiple active electrodes are used with the same reference, or vice versa. The notation (A1+A2) denotes linked earlobe electrodes. ND means no relevant data were found in the respective paper. The “Figure 2 number” column refers to the numbered panels in Figure 2 representing the effects of processing raw VEP data using the same frequency filter passbands. *—In these papers, the authors’ original nomenclature for active electrodes is employed. For the sake of uniformity, these electrodes have been renamed according to the 10–10 system designations [18] for electrodes located similarly. **—The 10–500 Hz passband is actually illustrated in Figure 2.

The variants of frequency filters used in intraoperative VEP monitoring are no less diverse (see Table 2) than the variants of the used derivations. Various frequency filter passbands have been documented, including those from 1–5 to 100 Hz (corresponding to the standard for clinical VEPs) [3,23,24,39,40], from 20 to 500 Hz [32,33,38], from 10 to 1000 Hz [26,29], from 2 to 400 Hz [34], from 0.1 to 200 Hz [30], from 1 to 300 Hz [35], from 10 to 100 Hz [25], and from 10 to 200 Hz [36]. In the case of VEPs, the choice of the optimal frequency filter passband, especially its lower limit, is a more complex task than for SSEP. This complexity arises because, as mentioned above, the length of VEP waves partially overlaps with that of the alpha rhythm of the background EEG. Since the characteristics of the VEPs, the alpha rhythm range, as well as their responses to anesthetics and existing pathologies can vary among patients, it is essential to select the passband of the frequency filter on an individual basis. In cases involving prechiasmal lateralized pathology, the optimal filter options may even differ between the eyes of the same patient. With regard to frequency filtering, we also utilized a personalized approach [37] simultaneously with the selection of reference electrodes. At the beginning of the surgery, after recording VEPs using different variants of reference electrode locations, we tried different frequency filtering options on all of them. The combinations of derivations and filters were selected based on the criteria of reproducibility, distinguishability, and amplitude of the VEP peaks. In the case of VEPs, simultaneous optimization by two parameters is important. Indeed, if more “aggressive” filtering is possible, the contribution of “EEG noise” to the SNR value will be significantly reduced, thereby favoring derivations that maximize VEP amplitude. If a “softer” filter is applied, then derivations with high background “EEG noise” may occur suboptimal even if the VEP amplitude is maximal there. The analysis of 240 operations revealed that the optimal frequency filter boundaries vary widely (Table 3). The most frequently utilized high-pass filter had a cutoff frequency of 10 Hz, whereas the predominant low-pass filter had a cutoff of 200 Hz; however, the 10–200 Hz passband was employed in less than half of the cases. Thus, similar to the derivation selection, it is impractical to propose a universally applicable frequency filter option. Thus, the justification for employing a personalized approach for frequency filter selection is also robust.

During VEP monitoring, the stimulation rate is limited mainly by the retinal adaptation time; the rate reported in most studies is about 1 Hz (see Table 2): as the frequency increases, the amplitude of the responses decreases. Moreover, in some cases, response exhaustion may be observed even at a stimulation rate of 1 Hz. In such a situation, paradoxically, reducing the stimulation frequency can lead to an acceleration of VEP acquisition by decreasing the required number of averages [37].

In addition to changing the stimulation frequency, other modifications of the delivered stimuli may be utilized in attempting to increase the amplitudes of the evoked responses, that is, the numerator of the SNR. One possible alternative approach for obtaining intraoperative VEPs involves the use of a rapid series of flashes, akin to the stimulus trains employed for eliciting intraoperative motor evoked potentials. Uribe et al. [38] investigated this method by applying a double flash with a 50 ms interval between flashes in patients undergoing spinal surgery. They compared the responses to a double flash with the responses to a standard single flash but did not report any differences. Alternatively, a technique for obtaining off-responses, i.e., reactions evoked by the cessation of light stimulation of the eye, was proposed for intraoperative VEP monitoring by A. Sato [30]. A pilot study involving 26 patients yielded promising results, indicating that off-responses were stable and obtainable for lower light intensities compared to standard on-responses. Furthermore, off-responses demonstrated adequate sensitivity to detect emerging visual dysfunctions during monitoring; they responded to manipulations of the optic nerve and exhibited recovery following the cessation of these manipulations. However, a subsequent study [41] on a larger cohort (134 eyes) did not reveal any significant advantages of off-responses over on-responses. Nevertheless, since off-responses showed better sensitivity at low light intensities, their application may be advantageous in scenarios where there is reason to suggest that the patient’s pupils are significantly constricted. This possibility requires further study.

**Figure 3:**
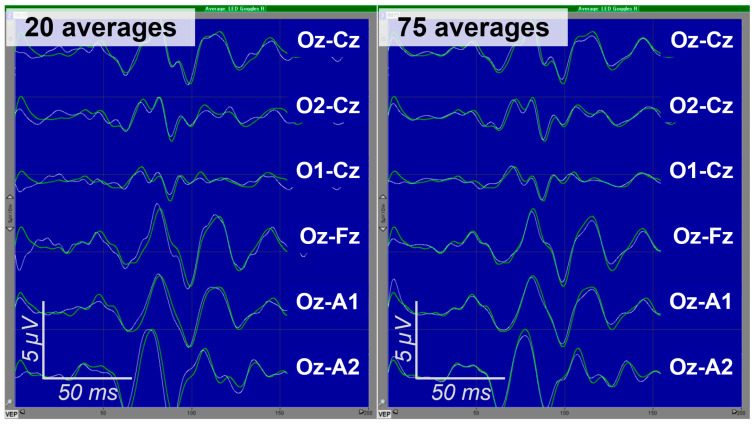
Example of excellently reproducible VEPs obtained using 15–300 Hz frequency filter. In both panels, baselines are represented by thicker green lines; they were recorded earlier with 100 averages. The thinner white lines represent results recorded with 20 (**left panel**) and 75 (**right panel**) averages under identical conditions, suggesting that there is no need to accumulate more averages after 20. Additionally, the between-derivation variability in VEP amplitudes is clearly observable. Adapted from [37] “Intraoperative monitoring of visual evoked potentials: experience of 240 operations”, by E.A. Levin, M.G. Kilchukov and A.A. Glushaeva, 2024, *Neyrokhirurgiya = Russian Journal of Neurosurgery*, *26*(3), p. 59, Figure 1c (https://doi.org/10.17650/1683-3295-2024-26-3-57-71). CC BY 4.0 (https://creativecommons.org/licenses/by/4.0/, accessed on 20 November 2024).

The issue of the personalized optimization of intraoperative VEP monitoring is still in its incipient stage; however, the potential for its application seems to be no less than for SSEPs. Indeed, the implementation of a personalized approach to VEP monitoring has enabled a significant reduction in the number of averages required, from the generally accepted 100 to 20–50 [37]. In a large number of cases, excellent reproducibility of VEPs was achieved with as few as 20 averages, as demonstrated in Figure 3. The personalized optimization of VEP monitoring deserves broader dissemination and should ideally be incorporated in clinical recommendations when they are developed.

## 5. Brainstem Acoustic Evoked Potentials

Unlike cortical SSEPs and VEPs, BAEPs reflect the activity of subcortical structures, that is, the brainstem nuclei (cochlear nucleus, peak III; superior olivary complex and lateral lemniscus, IV; lateral lemniscus and inferior colliculus, V) and the vestibulocochlear nerve (peaks I and II) [9]. The potentials of these structures, recorded on the surface of the head, have latencies and amplitudes that are an order of magnitude smaller than that of EPs of cortical origin. As a result, the number of averages required to record BAEPs reaches 500–2000 [4,42], which is partially compensated by the possibility of delivering stimuli at a high rate. Nevertheless, the BAEP recording duration can reach 2 min for each ear [4], and reducing it could significantly improve BAEP monitoring.

The recommended derivations for the intraoperative recording of BAEPs across the majority of the literature (e.g., [4,5,42,43]) are Mi–Cz and Mc–Cz (where M stands for mastoid processes). If the incision location precludes electrode placement on the mastoid, it is recommended to place it over the preauricular notch or on the ear lobe (Ai and Ac). As additional options, the Ai–Ac or Mi–Mc and Ncf–Cz (Ncf refers to noncephalic reference) derivations are also suggested [42,43]. The utilization of multiple derivations is a standard recommendation due to the fact that different BAEP waves are generated in different locations and are projected with maximum amplitude in distinct areas of the head surface. Specifically, while all principal peaks (I, III, and V) are usually recorded in standard derivations, Ai–Ac/Mi–Mc usually “specialize” in peak I and Ncf–Cz—in peak V. Regarding the latter, Greve et al. [44] demonstrated that the EPi–Cz derivation (EPi is the ipsilateral Erb’s point) provides a statistically significant increase in the amplitudes of peaks IV and V by approximately 50%. However, these authors cautioned that this enhancement in signal amplitude can be leveled out by an increase in the noise level. This consideration is particularly pertinent for the EPi–Cz derivation. Indeed, a higher level of myographic noise can be expected for EPi than for A1/A2/M1/M2, and surgeries involving BAEP monitoring are frequently conducted without muscle relaxation to allow for monitoring of motor evoked potentials and mapping the motor branches of the cranial nerves.

Regarding the frequency filtering during BAEP monitoring, clinical guidelines [4] and other publications [42,43] are also close to consensus: it is recommended to employ a cutoff frequency of 100–150 Hz for the HPF and 2500–3000 Hz for the LPF. A high cutoff for the HPF is possible since the interpeak intervals in BAEPs do not exceed 3–4 ms even in the case of prolongation due to impacts on the auditory nerve or tract [42]. As a result, the HPF effectively eliminates not only all high-amplitude oscillations of the background EEG but also network interference. At the same time, a filter with this passband permits significant transmission of myographic artifacts. This fact largely explains the optimality of using Cz, located remote from the cranial muscles, as a reference electrode. Conversely, Mi/Mc/Ai/Ac/Ncf leads are more susceptible to noise, resulting in a low single-trial SNR for BAEPs. To address this problem, a method for selecting the optimal filter for BAEP registration was proposed back in 1982 [6]. For this purpose, the recorded signal was subjected to Fourier decomposition, after which two indices were calculated and analyzed by comparing stimulus-locked epochs to epochs from background records devoid of stimuli for each frequency sub-band. The first index was spectral amplitude attenuation after increasing the number of averages from 200 to 2000. Attenuation in epochs without stimuli was large for the whole band, while for stimulus-locked averages it was low within the 450–1400 Hz sub-band. The second index was the mean phase variance across trials. And, again, the phase variance in the 450–1400 Hz sub-band was significantly lower for stimulus-locked trials. Based on these findings, the authors concluded that the components outside this sub-band represent noise and advocated for the use of a 450–1400 Hz bandpass filter. As a result, the good reproducibility of responses was achieved with 400 averages, which allowed for obtaining BAEPs in less than a minute even at the relatively low stimulation rate (10 Hz) employed in the study. In 1986, Hammerschlag et al. [45] reported the successful application of this method [6] for intraoperative BAEP monitoring. This time, the stimulation frequency was increased to 27.8 Hz, and only 5 to 10 s were required to obtain BAEPs, which enabled the authors to designate their approach as “real-time monitoring”. Experience of about 70 operations demonstrated the effectiveness of this method. In several cases, the detection of dysfunction via BAEPs was followed by changes in surgical maneuvers, resulting in subsequent signs of recovery; postoperative hearing was preserved in these cases. However, despite these results, this approach was not developed further. One potential explanation for this stagnation may be the excessive smoothing of the filtered BAEPs. In some cases, the morphology of consecutive waves may appear notably similar [6] (p. 411, Figure 5). Being unable to differentiate the waves by their appearance is rather uncomfortable; moreover, it bears a risk of missing the latency shift if this shift is close to the interval between the BAEP waves. Nonetheless, the bandpass filter optimization in BAEPs looks promising and deserves to be revisited.

Another approach to reducing the time required to obtain BAEPs may be to increase the stimulation rate. Indeed, normally, the monitored I–V waves fit into approximately 8 milliseconds; under the influence of existing pathology and/or anesthetics, the latencies may increase, but 15 msec will be sufficient in almost any case. Thus, the theoretical limit of stimulation frequency is about 70 Hz. In the literature, however, various stimulation frequencies are recommended–from 5 to 12 Hz [4] to 33.1 [43] and 10 to 40 [5] Hz. Similar to other sensory EPs, the amplitudes of BAEP peaks tend to decrease with increasing stimulation frequency, and the question is at what frequency lies the optimum in terms of the speed of obtaining sufficiently reproducible responses. Joo et al. [11] attempted to find it by analyzing the SNR at different a number of averages and stimulation frequencies. According to their data, the optimal frequency was 43.9 Hz, which facilitated the acquisition of well-reproducible BAEPs in less than 10 s with 400 averages. This result also allowed them to call the proposed technique “real-time monitoring”. It is noteworthy that these authors employed an unconventional frequency filter passband of 100–1000 Hz but did not justify its choice. This “aggressive” filtering may have contributed to their ability to achieve reproducible BAEP recording with a relatively low number of averages. In their study, the abovementioned stimulation frequency of 43.9 Hz, determined at the preliminary stage, was employed in all of the following 254 analyzed microvascular decompressions in patients with hemifacial spasms. Such patients typically do not exhibit hearing impairment prior to surgery; however, in patients with pre-existing hearing deficits, a lower stimulation frequency may be optimal. A method for the personalized selection of the rate of acoustic stimuli presentation is proposed in the review by Simon [5]: starting with a rate of 20 Hz and reducing it until high-quality BAEPs are obtained if they were not obtained for 20 Hz, or increasing the rate if high-amplitude, well-reproducible BAEPs were obtained initially. In this way, it is possible to achieve the fastest possible recording of high-quality BAEPs. Typically, according to Simon, the optimal stimulation frequency lies within the range between 10 and 40 Hz. Considering the findings of Joo et al. [11], it may be advantageous to initiate the stimulation rate selection from values of about 40 Hz, especially for patients with preserved hearing.

The BAEP stimulation technique using short broad-band clicks is well-established and effective. However, some advances in this field have emerged over the recent decade. One notable advancement is the introduction of chirp stimulation [46]. This technique addresses the unequal time required for sound waves of different frequencies to reach respective frequency-specific zones in the cochlea. According to this concept, when a broad-band click is decomposed into its frequency spectrum components by the cochlea, it becomes temporally “smeared” due to the aforementioned frequency-specific disparities in tympanum-to-basilar membrane delays. The time–frequency profile of a chirp stimulus is constructed to compensate for these unequal delays. Specifically, the stimulus wave train initiates with low-frequency oscillations, with the frequency gradually increasing towards the end of the wave packet. It is proposed that this approach activates all frequency-specific nerve fibers in the cochlear nerve synchronously, producing sharper and higher amplitude BAEP waves. Chirp stimulation was compared to standard broad-band clicks by Mastronardi et al. [47], who concluded that chirp-evoked acoustic brainstem responses are clearer and can be obtained more rapidly than responses to standard clicks. This characteristic renders chirp stimulation advantageous for monitoring cochlear nerve preservation during the surgical removal of vestibular schwannomas. Nonetheless, larger studies are necessary to gather sufficient evidence for the inclusion of this technique in commercial IONM equipment. Of note, had this happened, it is likely that chirp stimuli would require personalization to accommodate the individual characteristics of patients’ cochleae. Recently, the personalized optimization of intraoperative BAEP monitoring, which included the choice of the stimulating sound evoking the best responses, was presented in the work of Hosoya et al. [48]. Unfortunately, these authors did not provide sufficient details regarding their methodology.

The optimization of the intraoperative BAEP monitoring parameters is infrequently employed despite existing evidence indicating a substantial improvement in monitoring effectiveness associated with its implementation. Importantly, this improvement was demonstrated not only by a significant reduction in the duration of BAEP recording (this is an important index, but still indirect) but also by a statistically significant decrease in the incidence of iatrogenic hearing impairment after the optimization of intraoperative BAEP monitoring was implemented [11].

## 6. Problems in the Implementation of a Personalized Approach to Intraoperative Sensory EP Monitoring and Their Possible Solutions

One may question why the personalized optimization of intraoperative sensory EP monitoring, if it indeed increases the monitoring effectiveness, is not implemented more broadly. The likely explanation is that there are several factors impeding such implementation.

First, there is the factor of professional mindset. Both IONM technicians and supervising physicians are typically required to possess basic training or experience in “general” clinical neurophysiology [49]. Such training is usually focused on the use of neurophysiological methods in clinical diagnostics, fostering adherence to established standards when registering EPs (see Table 1). And, as, in most cases, it is actually possible to obtain EPs using these standards (albeit spending more time for it), practitioners may tend to persist in utilizing such an approach. Moreover, the mechanical transfer of methods for recording sensory EPs from neurophysiological clinical diagnostics to IONM is explicitly endorsed, for example, in the current clinical practice guidelines for IONM of the Korean Neurological Association [50]. This mindset can be addressed during IONM training by focusing on the differences between outpatient neurophysiological diagnostics and IONM and on the benefits of optimizing EP monitoring parameters. However, at present, only the most recent clinical guidelines for SSEP monitoring from ISIN [7] and ACNS [2] promote optimization, while the guidelines for BAEP monitoring issued earlier [4,9] do not address this aspect, and there are no specific guidelines for IONM for the VEPs at all. Nonetheless, recent progress in this direction is notable not only for SSEPs. For instance, in the recent revision of the standards and guidelines for the accreditation of educational programs in intraoperative neurophysiologic monitoring issued by the Commission on Accreditation of Allied Health Education Programs (CAAHEP) [51], the required competencies list includes not only the anesthesia parameter optimization for IONM (as in the previous revision) but also the signal optimization techniques.

Second, the implementation of a personalized approach to the intraoperative monitoring of sensory EPs may also be hampered by limitations of the existing IONM systems and their software. In the case of entry-level systems, which are relevant for low-income countries, the number of input channels may be limited. This limitation precludes the selection of the optimal combination of electrodes in the most convenient way of a direct comparison of simultaneously obtained responses. In this case, a sequential comparison may be employed by installing electrodes for all desired combinations on the patient, bringing their connectors to the contact box (which is usually accessible during surgery), and connecting them set by set. The software in some IONM systems allows for using only a fixed set of settings (in particular, options for the cutoff frequency of frequency filters) without the possibility of more flexible adaptation to specific conditions. More active interaction of neurophysiologists and neurophysiological societies with equipment manufacturers may facilitate the solution to this problem.

Third, personalized EP optimization may be quite time-consuming. While experienced practitioners typically possess the knowledge to navigate the optimization process effectively, novices may have to go through many variants before finding optimal one. In this context, collaboration within the entire neurosurgical team is essential. Early preparation is beneficial, and allowing for a brief delay before starting surgery can facilitate the recording of all EPs without interference. This way neurophysiologist usually can find optimal recording parameters without feeling excessive pressure. And striving for perfection should not hinder the main task. If, for example, one manages to find a combination of parameters that allow for obtaining well-reproducible SSEPs in 15 s, there is usually little reason to persist in searching for an option that takes 8 s to record them (even though it is 2 times faster). Anyway, the pauses in monitoring caused by the use of electrocautery are likely to be several times longer.

Last but certainly not least, some possible errors associated with sensory EP monitoring optimization may discourage practitioners from using it. For example, overly aggressive filtering may result in an artifact being misidentified as a genuine signal. As Betty L. Grundy put it: «Filtered artifact may appear pleasing to the eye, but it bears no relationship to neurological function» [52] (p. 562). This mistake is very dangerous, as it can lead to false negative interpretations and missing alarms with devastating consequences for a patient. A possible solution to mitigate this issue might be to conduct control recording with more averages and a wider filter passband to make sure that authentic EPs are being recorded (or at least to understand that a certain peak is an artifact) and then apply filtering. Another possible type of optimization-related false-negative monitoring result may occur because recording electrodes, when placed in non-standard (and sometimes standard, though) positions, may record some subcortical activity. For example, if Fz is placed too anteriorly during VEP recording, it may pick up an electroretinogram, with its later waves potentially being confused with early- and mid-latency VEP waves. When monitoring SSEPs, the subcortical N18 peak may, in some derivations, be “mixed” with a cortical N20 peak [53]. In such cases, dysfunction at a higher cortical level may remain undetected due to the masking effects of the subcortical component. The multi-derivation recording approach proposed above can help prevent such errors by increasing the likelihood of differentiating between cortical and subcortical signals. In the case of VEPs, it is recommended to additionally record ERG from each eye using needle electrodes placed near the outer corners of the eyes [32] or below the eyebrows [31]. This practice not only allows for controlling the stimuli delivery to retina but also aids in identifying the contamination of VEP with electroretinogram and helps avoid the use of derivations susceptible to such interference.

## 7. Perspectives

One of the significant challenges hampering the implementation of changes in IONM methods is the difficulty in collecting data that satisfy the criteria of evidence-based medicine. Indeed, the top of the pyramid of evidence is occupied by randomized controlled trials (RCTs) and systematic reviews of RCTs [54]. But, in the case of comparing different methodological approaches in IONM, this top can hardly be achieved due to conflict with ethical constraints. Indeed, within the RCT concept, it would be necessary to follow strictly some algorithm, in accordance with the group to which the patient was assigned, even if this algorithm is obviously not effective enough in this case. This is clearly unethical in a situation where it is possible to try modifications that could enhance efficacy. As an alternative that maintains a high level of evidence while circumventing ethical problems, the “virtual IONM” approach may be proposed. For this purpose, while conducting neuromonitoring in the usual manner, one would simultaneously record raw single-trial responses in all used derivations with precise timing of all important intraoperative events. The resultant dataset can then be processed offline in various ways, comparing them with each other in terms of the reproducibility of the EPs, the speed of their acquisition, sensitivity, and the speed of response to intraoperative events. A similar approach was employed by Jin et al. [55] to substantiate the superiority of their “slope measure” for identifying significant changes in SSEPs compared to traditional alarm criteria.

Actually, the reactions of EPs to intraoperative events and setting reporting thresholds for these reactions are important points that were not addressed in this review. Traditionally, a so-called 10–50 warning criterion is used in IONM; specifically, a warning is issued if a 10% latency delay or 50% amplitude decrease has occurred. However, this criterion is purely empirical and lacks justification. The optimization of EP monitoring facilitates a reduction in time intervals between consecutive EP measurements and enhances the signal-to-noise ratio (SNR) as well as the reproducibility of EPs. Therefore, smaller changes in EPs could be detected, and evidence suggests that reporting these changes can enhance surgical safety [56]. Furthermore, the “virtual IONM” approach discussed in the previous paragraph could be utilized to propose optimized warning criteria for sensory EP monitoring.

An important problem that prevents the widespread use of a personalized approach to selecting optimal parameters for monitoring sensory EPs is the time-consuming nature of this selection, particularly for less experienced neurophysiologists. In the conditions of time constraints typical for real-life monitoring, identifying the optimal parameters can be challenging. This difficulty may be alleviated through the development of software capable of automatically comparing various options for processing raw single-trial responses obtained in the “quiet” fragment of the recording. Such software could recommend parameters for EP recording and signal processing that maximize the SNR and/or minimize the time required to obtain reproducible responses. Development, certification, and integration in IONM systems of a module that suggests variants of personalized optimization for specific monitoring sessions could confer a competitive advantage to certain manufacturers in the IONM sector.

## 8. Conclusions

Summing up, the IONM community has accumulated some evidence in favor of the personalized optimization of intraoperative monitoring parameters for each of the sensory EP modalities and some practical experience in applying this approach. However, as of now, efforts to introduce personalized optimization into widespread practice are primarily observed mostly for SSEPs. The most recent clinical guidelines [2,7] on intraoperative SSEP monitoring use the concept of “optimization” for this purpose, which actually means a personalized selection of stimulation and registration parameters for SSEPs. As for other sensory modalities, this approach is not only missed in the guidelines but is discussed only in isolated papers on monitoring BAEPs [5,11,45] and VEPs [37,57]. Unfortunately, the nature of IONM, which is both a diagnostic method and one that affects surgical treatment and its outcomes, makes it difficult to conduct randomized trials that meet the standards of evidence-based medicine. However, the possibility of conducting research in the “virtual IONM” format may facilitate the accumulation of more convincing data than retrospective series and support a broader implementation of the personalized approach to sensory EP monitoring. Such implementation—including the adaptation of IONM equipment and software to it—has the potential to accelerate the acquisition of these EPs during surgical procedures, thereby advancing towards true real-time monitoring. In turn, providing surgeons with prompt information regarding disturbances in controlled sensory functions will facilitate the achievement of surgical objectives while maximizing patient safety.

## Figures and Tables

**Table 1:** Differences between evoked potential recording during clinical neurophysiological diagnostics and during their intraoperative monitoring.

	Recording of EPs During Clinical Neurophysiological Diagnostics	Intraoperative Monitoring of EPs
Task	Identification of *existing* sensory impairments	Detection of *emerging new* sensory dysfunctions
Dysfunction detection method	Comparison with *the population norm*	Comparison with *the same patient’s records* obtained at the beginning of the operation
Conditions	Patient is *conscious*; *external interference is minimized*; *electromyographic interference* may be significant	Patient is *under general anesthesia*; *external interference* (electrocoagulation, mechanical, etc.) *is usual*; *electromyographic interference* can be reduced by muscle *relaxation*
Registration parameters	Same as for the normative population, *same for all patients*	Changes during monitoring are undesirable, but *differences between patients are acceptable*
Time limits	*No* *significant time limitations*	It is necessary *to update records as often as possible*

**Table 3:** Low- and high-pass filters selected for intraoperative VEP monitoring in the group of patients analyzed in Levin et al., 2024 [37].

High-Pass Filter, Hz	The Number of Eyes for Which VEPs Were Recorded Using the Specified Frequency Filters, n						
	Low-Pass Filter, Hz						Total
	100	150	200	250	300	350–400	
5	1	6	29	1	0	0	**37**
10	2	47	196	13	26	2	**286**
15	2	4	74	6	7	1	**94**
20	0	2	1	0	0	1	**4**
**Total**	**5**	**59**	**300**	**20**	**33**	**4**	**421**

Data on 480 eyes of 240 patients were analyzed. At the beginning of the monitoring session, the filter passband was selected for each eye based on the criteria of reproducibility and the amplitudes of main VEP peaks. In some cases, different filter settings were applied for the left and right eyes. The numbers in each cell indicate the quantity of eyes monitored with the corresponding LPF and HPF combination. In 59 of 480 eyes, VEPs were unobtainable (most of them had visual dysfunctions preoperatively); these eyes are not included in this table. Of note, the 10 Hz HPF cutoff and 200 Hz LPF cutoff were utilized much more frequently than any other settings, but their combination, i.e., the 10–200 Hz passband, covered less than 50% of the eyes. From [37] “Intraoperative monitoring of visual evoked potentials: experience of 240 operations”, by E.A. Levin, M.G. Kilchukov and A.A. Glushaeva, 2024, *Neyrokhirurgiya = Russian Journal of Neurosurgery*, *26* (3), p. 61, Table 1 (https://doi.org/10.17650/1683-3295-2024-26-3-57-71). CC BY 4.0 (https://creativecommons.org/licenses/by/4.0/, accessed on 20 November 2024).

## Data Availability

Not applicable.

## References

[1] Raudzens P.A. (1982). Intraoperative monitoring of evoked potentials. Ann. N. Y. Acad. Sci..

[2] Toleikis J.R., Pace C., Jahangiri F.R., Hemmer L.B., Toleikis S.C. (2024). Intraoperative somatosensory evoked potential (SEP) monitoring: An updated position statement by the American Society of Neurophysiological Monitoring. J. Clin. Monit. Comput..

[3] Luo Y., Regli L., Bozinov O., Sarnthein J. (2015). Clinical utility and limitations of intraoperative monitoring of visual evoked potentials. PLoS ONE.

[4] American Clinical Neurophysiology Society Guideline 11C: Recommended Standards for Intraoperative Monitoring of Auditory Evoked Potentials. https://www.acns.org/pdf/guidelines/Guideline-11C.pdf.

[5] Simon M.V. (2011). Neurophysiologic intraoperative monitoring of the vestibulocochlear nerve. J. Clin. Neurophysiol..

[6] Fridman J., John E.R., Bergelson M., Kaiser J.B., Baird H.W. (1982). Application of digital filtering and automatic peak detection to brain stem auditory evoked potential. Electroencephalogr. Clin. Neurophysiol..

[7] MacDonald D.B. (2001). Individually optimizing posterior tibial somatosensory evoked potential P37 scalp derivations for intraoperative monitoring. J. Clin. Neurophysiol..

[8] Scherg M., Berg P., Nakasato N., Beniczky S. (2019). Taking the EEG back into the brain: The power of multiple discrete sources. Front. Neurol..

[9] Martin W.H., Stecker M.M. (2008). ASNM Position Statement: Intraoperative monitoring of auditory evoked potentials. J. Clin. Monit. Comput..

[10] Odom J.V., Bach M., Brigell M., Holder G.E., McCulloch D.L., Mizota A., Tormene A.P. (2016). ISCEV standard for clinical visual evoked potentials: (2016 update). Doc. Ophthalmol..

[11] Joo B.-E., Park S.-K., Cho K.-R., Kong D.-S., Seo D.-W., Park K. (2016). Real-time intraoperative monitoring of brainstem auditory evoked potentials during microvascular decompression for hemifacial spasm. J. Neurosurg..

[12] Dimakopoulos V., Selmin G., Regli L., Sarnthein J. (2023). Optimization of signal-to-noise ratio in short-duration SEP recordings by variation of stimulation rate. Clin. Neurophysiol..

[13] Stecker M.M. (2000). Generalized averaging and noise levels in evoked responses. Comput. Biol. Med..

[14] MacDonald D.B., Dong C., Quatrale R., Sala F., Skinner S., Soto F., Szelényi A. (2019). Recommendations of the International Society of Intraoperative Neurophysiology for intraoperative somatosensory evoked potentials. Clin. Neurophysiol..

[15] MacDonald D.B., Zayed Z.A., Stigsby B. (2005). Tibial somatosensory evoked potential intraoperative monitoring: Recommendations based on signal to noise ratio analysis of popliteal fossa, optimized P37, standard P37, and P31 potentials. Clin. Neurophysiol..

[16] MacDonald D.B., Al-Zayed Z., Stigsby B., Al-Homoud I. (2009). Median somatosensory evoked potential intraoperative monitoring: Recommendations based on signal-to-noise ratio analysis. Clin. Neurophysiol..

[17] MacDonald D.B. (2020). Monitoring somatosensory evoked potentials. Neurophysiology in Neurosurgery.

[18] Nuwer M.R., Comi G., Emerson R., Fuglsang-Frederiksen A., Guérit J.-M., Hinrichs H., Ikeda A., Luccas F.J.C., Rappelsburger P. (1998). IFCN standards for digital recording of clinical EEG. Electroencephalogr. Clin. Neurophysiol..

[19] Dietrich C., Blume K.R., Franz M., Huonker R., Carl M., Preißler S., Hofmann G.O., Miltner W.H.R., Weiss T. (2017). Dermatomal organization of SI leg representation in humans: Revising the somatosensory homunculus. Cereb. Cortex.

[20] Hanson C., Lolis A.M., Beric A. (2016). SEP montage variability comparison during intraoperative neurophysiologic monitoring. Front. Neurol..

[21] De Weerd J.P.C., Kap J.I. (1981). Spectro-temporal representations and time-varying spectra of evoked potentials. Biol. Cybern..

[22] Allison D.W., Silverstein J.W., Thirumalai S.S., D’Amico R.S. (2022). Misconceptions in IONM Part III: Stimulation repetition rate effects on intraoperative somatosensory evoked potential amplitude and latency. Neurodiagn. J..

[23] Shcherbuk A.Y., Shcherbuk Y.A., Pyanzin S.Y. (2011). Intraoperative monitoring of visual evoked potentials as a component of a comprehensive neurophysiological supply of transnasal endoscopic interventions for tumors of chiasmatic-sellar region. Ross. Neirokhirurgicheskii Zhurnal Im. Prof. A.L. Polenova = Russ. J. Neurosurg. n. A. Prof. A.L. Polenov.

[24] Houlden D.A., Turgeon C.A., Polis T., Sinclair J., Coupland S., Bourque P., Corsten M., Kassam A. (2013). Intraoperative flash VEPs are reproducible in the presence of low amplitude EEG. J. Clin. Monit. Comput..

[25] Houlden D.A., Turgeon C.A., Amyot N.S., Edem I., Sinclair J., Agbi C., Polis T., Alkherayf F. (2019). Intraoperative flash visual evoked potential recording and relationship to visual outcome. Can. J. Neurol. Sci./J. Can. Des Sci. Neurol..

[26] Kodama K., Goto T. (2020). Neurophysiology of the visual system: Basics and intraoperative neurophysiology techniques. Neurophysiology in Neurosurgery.

[27] Vijayan S., Ching S., Purdon P.L., Brown E.N., Kopell N.J. (2013). Thalamocortical Mechanisms for the Anteriorization of Alpha Rhythms during Propofol-Induced Unconsciousness. J. Neurosci..

[28] Blain-Moraes S., Tarnal V., Vanini G., Alexander A., Rosen D., Shortal B., Janke E., Mashour G.A. (2014). Neurophysiological correlates of sevoflurane-induced unconsciousness. Anesthesiology.

[29] Kodama K., Goto T., Sato A., Sakai K., Tanaka Y., Hongo K. (2010). Standard and limitation of intraoperative monitoring of the visual evoked potential. Acta Neurochir..

[30] Sato A. (2016). Interpretation of the causes of instability of flash visual evoked potentials in intraoperative monitoring and proposal of a recording method for reliable functional monitoring of visual evoked potentials using a light-emitting device. J. Neurosurg..

[31] Nakagawa I., Park H., Kotsugi M., Yokoyama S., Omoto K., Myochin K., Takeshima Y., Matsuda R., Nishimura F., Yamada S. (2022). Diagnostic impact of monitoring visual evoked potentials to prevent visual complications during endovascular treatment for intracranial aneurysm. Front. Neurol..

[32] Sasaki T., Itakura T., Suzuki K., Kasuya H., Munakata R., Muramatsu H., Ichikawa T., Sato T., Endo Y., Sakuma J. (2010). Intraoperative monitoring of visual evoked potential: Introduction of a clinically useful method. J. Neurosurg..

[33] Miyagishima T., Tosaka M., Yamaguchi R., Nagaki T., Ishii N., Kojima T., Yoshimoto Y. (2019). Extended endoscopic endonasal resection of craniopharyngioma using intraoperative visual evoked potential monitoring: Technical note. Acta Neurochir..

[34] Gutzwiller E.M., Cabrilo I., Radovanovic I., Schaller K., Boëx C. (2018). Intraoperative monitoring with visual evoked potentials for brain surgeries. J. Neurosurg..

[35] Hariharan P., Balzer J.R., Anetakis K., Crammond D.J., Thirumala P.D. (2017). Electrophysiology of olfactory and optic nerve in outpatient and intraoperative settings. J. Clin. Neurophysiol..

[36] Olmsted Z.T., Silverstein J.W., Einstein E.H., Sowulewski J., Nelson P., Boockvar J.A., D’Amico R.S. (2023). Evolution of flash visual evoked potentials to monitor visual pathway integrity during tumor resection: Illustrative cases and literature review. Neurosurg. Rev..

[37] Levin E.A., Kilchukov M.G., Glushaeva A.A. (2024). Intraoperative monitoring of visual evoked potentials: Experience of 240 operations. Russ. J. Neurosurg..

[38] Uribe A.A., Mendel E., Peters Z.A., Shneker B.F., Abdel-Rasoul M., Bergese S.D. (2017). Comparison of visual evoked potential monitoring during spine surgeries under total intravenous anesthesia versus balanced general anesthesia. Clin. Neurophysiol..

[39] Tao X., Fan X., Gui S., Liu J., Yang X., Li K., Yang J., Li C., Qiao H. (2023). Efficacy of intraoperative visual evoked potential amplitude reduction in predicting visual outcome after extended endoscopic endonasal resection of craniopharyngiomas. J. Neurosurg..

[40] San-Juan D., Cortés M.E., Tena-Suck M., Garduño A.J.O., Pizano J.A.L., Domínguez J.V., Gónzalez-Aragón M.F., Gómez-Amador J.L. (2016). Neurophysiological intraoperative monitoring during an optic nerve schwannoma removal. J. Clin. Monit. Comput..

[41] Hardian R.F., Ogiwara T., Sato A., Fujii Y., Suzuki Y., Hanaoka Y., Miyata M., Kamiya K., Sasaki T., Goto T. (2021). Comparison between conventional flash and Off-Response intraoperative visual evoked potential monitoring for endoscopic endonasal surgery. Oper. Neurosurg..

[42] Seubert C.N., Herman M., Koht A., Sloan T., Toleikis J. (2017). Auditory-Evoked potentials. Monitoring the Nervous System for Anesthesiologists and Other Health Care Professionals.

[43] Zamel K.M. (2010). Brainstem auditory evoked potential monitoring. Intraoperative Neurophysiologic Monitoring.

[44] Greve T., Beyer F., Szelényi A. (2019). Intraoperative Erb’s Point-Vertex recording increases brainstem auditory evoked potential wave V amplitude. Clin. Neurophysiol..

[45] Hammerschlag P.E., Berg H.M., Prichep L.S., John E.R., Cohen N.L., Ransohoff J. (1986). Real-Time Monitoring of Brainstem Auditory Evoked Response (BAER) during Cerebellopontine Angle (CPA) Surgery. Otolaryngology.

[46] Di Scipio E., Mastronardi L. (2015). CE-Chirp^®^ ABR in cerebellopontine angle surgery neuromonitoring: Technical assessment in four cases. Neurosurg. Rev..

[47] Mastronardi L., Di Scipio E., Cacciotti G., Roperto R., Scavo C.G. (2018). Hearing preservation after removal of small vestibular schwannomas by retrosigmoid approach: Comparison of two different ABR neuromonitoring techniques. Acta Neurochir..

[48] Hosoya M., Nishiyama T., Wakabayashi T., Shimanuki M.N., Miyazaki H., Ozawa H., Oishi N. (2023). Vestibular Schwannoma Surgery with Endoscope-Assisted Retrolabyrinthine Approach under Modified Reinforced Continuous Intraoperative Monitoring for Hearing Preservation: Experience of 33 Cases in a Single Center. Diagnostics.

[49] Nuwer M.R., Galloway G.M., Nuwer M.R., Lopez J.R., Zamel K.M. (2010). Introduction, history, and staffing for intraoperative monitoring. Intraoperative Neurophysiologic Monitoring.

[50] (2021). Clinical practice guidelines for intraoperative neurophysiological monitoring: 2020 update. Ann. Clin. Neurophysiol..

[51] Commission on Accreditation of Allied Health Education Programs Standards and Guidelines for the Accreditation of Educational Programs in Intraoperative Neurophysiologic Monitoring. https://cdn.prod.website-files.com/5f466098572bfe97f28d59df/614b674da83bc04a1fab718a_IONM_Standards_Approve3192021.pdf.

[52] Grundy B.L. (1982). Monitoring of sensory evoked potentials during neurosurgical operations: Methods and applications. Neurosurgery.

[53] Fried S.J., Smith D.M., Legatt A.D. (2014). Median nerve somatosensory evoked potential monitoring during carotid endarterectomy. J. Clin. Neurophysiol..

[54] Burns P.B., Rohrich R.J., Chung K.C. (2011). The levels of evidence and their role in Evidence-Based Medicine. Plast. Reconstr. Surg..

[55] Jin S.-H., Chung C.K., Kim J.E., Choi Y.D. (2014). A new measure for monitoring intraoperative somatosensory evoked potentials. J. Korean Neurosurg. Soc..

[56] Park S.-K., Lee H.S., Cho K.R., Park K. (2023). Recent advances in intraoperative brainstem auditory evoked potential monitoring during microvascular decompression surgery for hemifacial spasm. Life.

[57] Rajan S., Simon M.V., Nair D. (2016). Intraoperative visual evoked potentials: There is more to it than meets the eye. J. Neurol. Neurosci..

